# High-energy synchrotron X-ray multimodal computed tomography: enabling multiscale materials characterization at NSLS-II

**DOI:** 10.1107/S1600577526001104

**Published:** 2026-02-19

**Authors:** Mehmet Topsakal, Daniel O’Nolan, Michael Drakopoulos, Jianming Bai, Eric Dooryhee, Simerjeet K. Gill, Sanjit Ghose

**Affiliations:** a Nuclear Science and Security Brookhaven National Laboratory Upton NY11973 USA; b RTI International Research Triangle Park NC27709 USA; c National Synchrotron Light Source II Brookhaven National Laboratory Upton NY11973 USA; d Univ. Grenoble Alpes, CNRS, Grenoble INP (Institute of Engineering), Institut Néel, 38000Grenoble, France; Australian Synchrotron, Australia

**Keywords:** X-ray imaging, X-ray diffraction, X-ray fluorescence, total scattering, computed tomography, nuclear materials

## Abstract

We report the commissioning of a multimodal computed tomography experimental setup at the 28-ID-2 (XPD) beamline of the National Synchrotron Light Source II. This high-energy (>60 keV) resource features a tunable X-ray beam size ranging from several millimetres to a few micrometres and enables comprehensive characterization of high-*Z* materials—an essential capability for nuclear and advanced materials research. It provides four complementary computed tomography modalities: X-ray absorption, X-ray fluorescence, X-ray diffraction, and pair distribution function tomography. A case study using a custom-made heterogeneous sample demonstrates these abilities to simultaneously capture atomic, elemental, and morphological information. This unique combination of imaging, structural, and chemical sensitive methods provides a holistic approach to study complex materials with amorphous and crystalline systems across multiple length scales.

## Abstract 2

The development of multimodal X-ray computed tomography capabilities, including imaging, fluorescence, diffraction, and scattering, at the 28-ID-2 beamline of the National Synchrotron Light Source II is described.

## 1. Introduction

Beamline 28-ID-2 (XPD) at the National Synchrotron Light Source II (NSLS-II) is a high energy X-ray powder diffraction beamline designed for advanced X-ray based synchrotron studies of materials (Shi *et al.*, 2013 ▸). It delivers hard X-rays to probe bulk materials and materials in complex sample environments, including sealed cells and functional devices such as batteries. This capability enables *in situ* and *operando* studies across diverse research fields ranging from physics, chemistry, and materials science, to earth and environmental sciences, and engineering. The beamline is optimized to monitor and characterize the structural evolution of materials under non-ambient or dynamic conditions as a function of temperature, pressure, pH, reactive gases, and applied electric or magnetic field (Zhang *et al.*, 2025 ▸; Liu *et al.*, 2025 ▸). Furthermore, the continued developments implement the integration of complementary experimental tools, versatile sample environments, and time-resolved data acquisition strategies to facilitate a comprehensive understanding of complex materials synthesis and processing pathways.

In recent years, there has been a growing demand among users for X-ray tomographic techniques to investigate the three-dimensional (3D) structure and morphology of materials (Omori *et al.*, 2023 ▸; Atwood *et al.*, 2015 ▸). These methods enable non-destructive visualization of a sample’s internal structure under native conditions, providing critical insights into its spatially resolved crystallographic and chemical features. Monitoring compositional and structural changes with spatial sensitivity allows the identification of distinct regions within heterogeneous systems, particularly where variations in density or composition occur. These methods enable non-destructive visualization of a sample’s internal structure under native conditions, providing critical insights into its spatially resolved crystallographic and chemical features. Monitoring compositional and structural changes with spatial sensitivity allows the identification of distinct regions within heterogeneous systems, particularly where variations in density or composition occur.

The integration of X-ray computed tomography with X-ray diffraction and scattering techniques enables simultaneous multi-scale characterization of hierarchical structures from the atomic (Å) to the macroscopic (mm) length scales. Among such methods, X-ray diffraction computed tomography (XRD-CT) (Harding *et al.*, 1987 ▸) has emerged as a promising *in situ* and *operando* approach for elucidating structural evolution in complex functional materials, including catalysts (Vamvakeros *et al.*, 2016 ▸), fuel cells (Li *et al.*, 2019 ▸), batteries (Matras *et al.*, 2022 ▸), as well as biological materials (Seknazi *et al.*, 2020 ▸), and applications in cultural heritage (Possenti *et al.*, 2024 ▸). Although pair distribution function computed tomography (PDF-CT) was first demonstrated conceptually in 2013 (Jacques *et al.*, 2013 ▸), its adoption has been limited due to the extended data collection times required to obtain high quality scattering data suitable for pair distribution function (PDF) analysis (Jensen *et al.*, 2022 ▸). Indeed, small angle X-ray scattering computed tomography has found success in studying heterogeneous materials (Schaff *et al.*, 2015 ▸; Liebi *et al.*, 2015 ▸). These CT techniques, which concern diffraction or scattering in general, often fall under the term diffraction/scattering computed tomography (DS-CT) (Birkbak *et al.*, 2015 ▸).

Here, we report the successful commissioning and implementation of all these tomographic techniques: X-ray absorption computed tomography (X-CT), X-ray fluorescence computed tomography (XRF-CT), XRD-CT, and PDF-CT at beamline 28-ID-2 of NSLS-II. The focus is on enabling fast, routine, and multi-scale acquisition of actionable, high-quality tomographic data for a broader user community. For a better understanding of all these techniques, we present a case study of a heterogeneous demonstration sample consisting of intertwined metal and oxide components, highlighting the utility of multimodal CT methods for probing complex and heterogeneous materials. We anticipate that results of this study will serve as valuable and resourceful reference for prospective NSLS-II users seeking to exploit the advanced tomographic capabilities available at the 28-ID-2 beamline that can be applied to a wide range of materials research in extreme environments, including, but not limited to, catalysis, batteries, nuclear reactors, and nuclear forensics.

## 2. Components of the instrument

**Figure 1:**
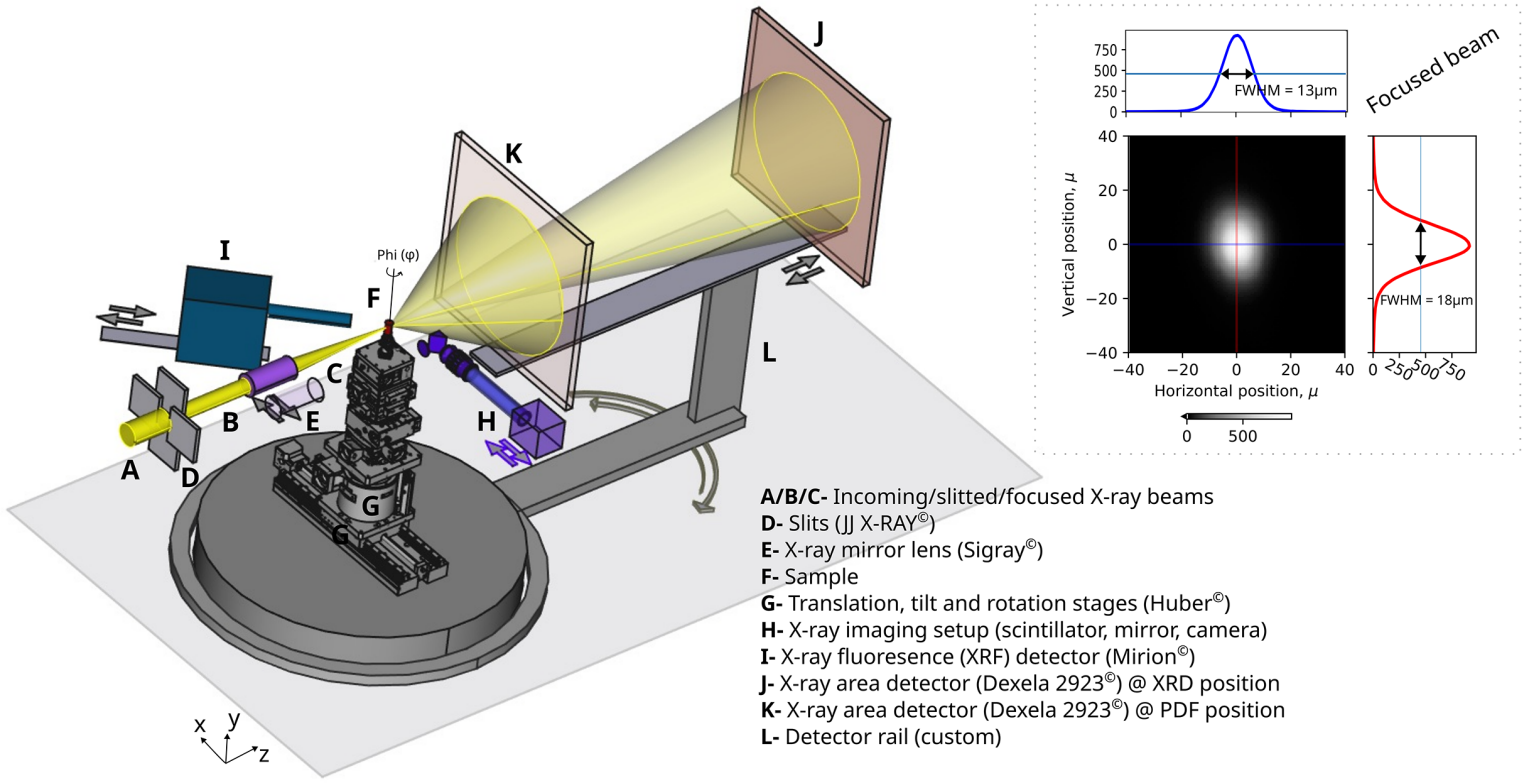
CAD-layout of the 28-ID-2-D beamline endstation (D-hutch) tomography instrument with major components highlighted by A to L. Arrows emphasize the freedom in the locations of the components. The measured vertical and horizontal beam profiles of the focused spot are shown in the upper right panel.

Beamline 28-ID-2 (XPD) at the NSLS-II is a high-energy wiggler beamline that delivers a tunable monochromatic X-ray beam in the energy range 30–70 keV. The beamline comprises two experimental hutches, designated C and D. The C-hutch serves as the primary experimental station and is equipped with instruments optimized for conventional XRD and PDF measurements. The nominal beam size in the C-hutch used for routine powder diffraction experiments is approximately 0.5 mm × 0.5 mm. The tomography instrument (Fig. 1 ▸) is located in the D-hutch, positioned downstream of the C-hutch, and is dedicated to high-energy X-ray computed tomography applications. The specifications, commercial suppliers and positions of the tomography instruments in D-hutch are listed in Table 1 ▸.

### 2.1. X-ray beam

The XPD beamline provides not only flexibility in energy tuning but also flexible control over the beam size in both horizontal and vertical directions. Horizontal focusing is achieved through a sagittally bent double Laue monochromator (DLM) (Zhong *et al.*, 2001*a* ▸; Zhong *et al.*, 2001*b* ▸), while vertical focusing is controlled by a large vertically bent mirror. By unbending both the DLM crystals and retracting the vertical focusing mirror from the beam path, an unfocused and nearly uniform X-ray beam of approximately 10 mm (H) × 2 mm (V) can be produced. Although such a large beam is not suitable for standard transmission-mode XRD or PDF experiments, it is ideal for X-ray imaging (full-field) applications. The incoming beam entering the instrument in D-hutch is indicated as position ‘A’ in Fig. 1 ▸.

A high precision slit system (JJ X-RAY, Denmark) equipped with 10 mm thick tungsten blades allows fine adjustment of the beam size. For XRF-CT, XRD-CT, and PDF-CT modes of operation, a platinum (Pt) coated achromatic ellipsoidal X-ray mirror lens (Sigray Inc., USA) with capillary-like geometry is employed to focus an 80 µm × 100 µm incident slitted beam down to approximately 13 µm × 18 µm (H × V), yielding a photon flux increase by a factor of 32 relative to the unfocused configuration. The mirror lens length is 150 mm with 0.63 mm entrance and 0.42 mm exit aperture diameters. The measured vertical and horizontal beam profiles of the focused spot are shown in the upper right panel of Fig. 1 ▸. The lens has a working distance of 110 mm and a convergence angle of less than 1 µrad. Partial lens illumination through upstream slits was necessary to achieve lower convergence angle. Furthermore, the lens assembly can be translated along both horizontal and vertical axes, enabling a rapid (<1 min) switch between large beam and micro-focused beam modes. The positions labeled B, C, D, and E in Fig. 1 ▸ correspond respectively to the slitted beam, focused beam, slit system, and X-ray lens.

### 2.2. Sample stage

Rotation, translation, and tilt stages are essential components of a CT setup. For illustrative purposes, a cylindrical sample (F, Fig. 1 ▸) is placed on a goniometer stack (HUBER Diffraktionstechnik GmbH, Germany) (G, Fig. 1 ▸). The current sample stage has a range of 100 mm in the horizontal translation with a maximum speed of 0.25 mm s^−1^ and 360° rotation with a maximum speed of 10° s^−1^. The topmost stage can carry samples up to 1 kg. Both translation and rotation stages support step-scan and fly-scan modes of operation whereas the latter is more suitable for time consuming CT experiments.

### 2.3. X-ray imaging component

An X-ray imaging system was developed in-house, consisting of (i) X-ray sensitive scintillator, (ii) right-angle mirror, (iii) microscope objective, (iv) extension tube, and (v) a digital visible-light sensor. The system configuration is illustrated in Fig. 1 ▸(H) and is mounted on a motorized stage to allow rapid activation or deactivation of the X-ray imaging mode within less than one minute. The scintillator employed is a cerium-doped gadolinium aluminium gallium garnet (GAGG:Ce) crystal scintillator (Crytur Inc., USA) of thickness 20 µm. The visible photons generated in the scintillator are redirected towards a 5× magnification microscope objective (Plan APO infinity-corrected, Edmund Optics, USA) using an aluminium-coated right-angle prism mirror. The objective features a numerical aperture (NA) of 0.14, a working distance of 34 mm, and a theoretical Rayleigh resolution limit of 2 µm. The visible-light image is captured using a high-speed digital camera (Emergent Vision Technologies, USA) that has a 3.45 µm × 3.45 µm CMOS sensor pixel size and with a maximum frame rate of 80 Hz. The detector provides a native resolution of 4096 × 3000 pixels, resulting in a 2.83 mm (H) × 2.07 mm (V) field of view and 0.69 µm × 0.69 µm effective voxel-size on the scintillator plane. The system supports a 12-bit digital conversion depth, ensuring high dynamic range for tomographic and radiographic measurements.

### 2.4. X-ray fluorescence component

An energy-dispersive high-purity germanium (HPGe) detector (Canberra Industries, now Mirion Technologies, USA) is used to collect X-ray fluorescence (XRF) signals for elemental identification. The detector is placed at 45° relative to the incident X-ray beam, as shown in Fig. 1 ▸(I), and operates effectively within an energy range of 2–100 keV. The germanium crystal has a thickness of 6 mm and an active diameter of 10 mm, with a carbon-based entrance window to minimize absorption losses. Both the detector crystal and the first-stage amplifier are cryogenically cooled to ensure optimal performance and stability. The system achieves an energy resolution better than 400 eV (FWHM) at 60 keV. Setting the energy of the incoming beam to 70 keV enables resolving *K* fluorescence lines from elements up to tungsten (*Z* = 74) and *L* lines from elements up to americium (*Z* = 95) and beyond, thereby providing broad elemental detection coverage across the periodic table.

### 2.5. XRD and PDF components

Diffraction and scattering measurements are performed using a CMOS-based X-ray area detector Dexela 2923 (Varex Imaging, USA) (Konstantinidis *et al.*, 2012 ▸). The detector is mounted on a custom-designed motorized rail system as shown in Figs. 1 ▸(J), 1(K), and 1(L). This configuration allows for precise control of both sample-to-detector distance and the horizontal angle of the detector relative to the incident beam. Sample-to-detector distance can be varied continuously from 100 mm to 1500 mm, while the detector angle can be adjusted within a range of ±25°. The detector features a pixel array of 3888 × 3072 with 75 µm × 75 µm pixel size, providing an active detection area of approximately 29 cm × 23 cm. It supports a maximum acquisition rate of 26 frames s^−1^ and a 14-bit digital dynamic range, enabling high-fidelity data collection for both diffraction and total scattering experiments.

### 2.6. Data acquisition and analysis

The data acquisition and analysis workflow for the tomography instrument integrates multiple software frameworks to ensure efficient and reproducible operation. Hardware communication and device coordination are managed through the Experimental Physics and Industrial Control System (EPICS: https://epics-controls.org/), which provides a robust infrastructure for low-level control. User-assisted experimental control and data collection are implemented via the Ophyd Python library (Arkilic *et al.*, 2017 ▸), while high-level beamline controls are carried out through Controls System Studio (CSS, https://controlsystemstudio.org/) that provides a graphical user interface (GUI) for instrument control and monitoring.

Upon completion of a full tomographic scan, multimodal raw data acquired from various detectors together with relevant experimental metadata such as timestamps, motor positions and user annotations are compiled into a single, self-describing, machine-independent NetCDF (Network Common Data Form) file. Data formatting and storage are handled using the *xarray* Python library (https://xarray.dev/). The resulting NetCDF files are subsequently transferred to large-scale network storage accessible to users via Globus (https://www.globus.org/).

A dedicated data analysis pipeline has been developed using *pyFAI* (Kieffer & Wright, 2013 ▸) and *tomopy* (Gürsoy *et al.*, 2014 ▸) Python libraries. The *pyFAI* package performs azimuthal integration of two-dimensional diffraction data into one-dimensional intensity profiles following dark-field subtraction and masking. Tomographic reconstructions are mainly carried out using *tomopy*, while the *Algotom* library (Vo *et al.*, 2021 ▸) provides advanced functionality for artifact removal, post-processing, data alignment, and reconstructions. X-ray fluorescence (XRF) data processing is conducted using the *XrayDB* Python library (https://xraypy.github.io/XrayDB/) enabling elemental identification and quantification.

## 3. Commissioning results and discussions

With the secondary focusing optics retracted from the beam path, X-CT was performed using a broad, unfocused beam. Under these conditions, a field of view (FOV) of 2.83 mm × 2.07 mm was achieved, though that was limited by the camera sensor dimensions. When the secondary focusing element was introduced into the beam path, a focused beam of 13 µm × 18 µm (H × V) was obtained (Fig. 1 ▸), which determined the effective voxel size for the XRD-CT and PDF-CT experiments.

In this section, we present the results of the commissioning experiments that assess the overall performance of the instrument for four complimentary modalities: X-CT, XRF-CT, XRD-CT, and PDF-CT. These methods were applied to a highly heterogeneous test specimen to evaluate imaging resolution, chemical sensitivity, and structural fidelity of the setup.

### 3.1. Demonstration sample

**Figure 2:**
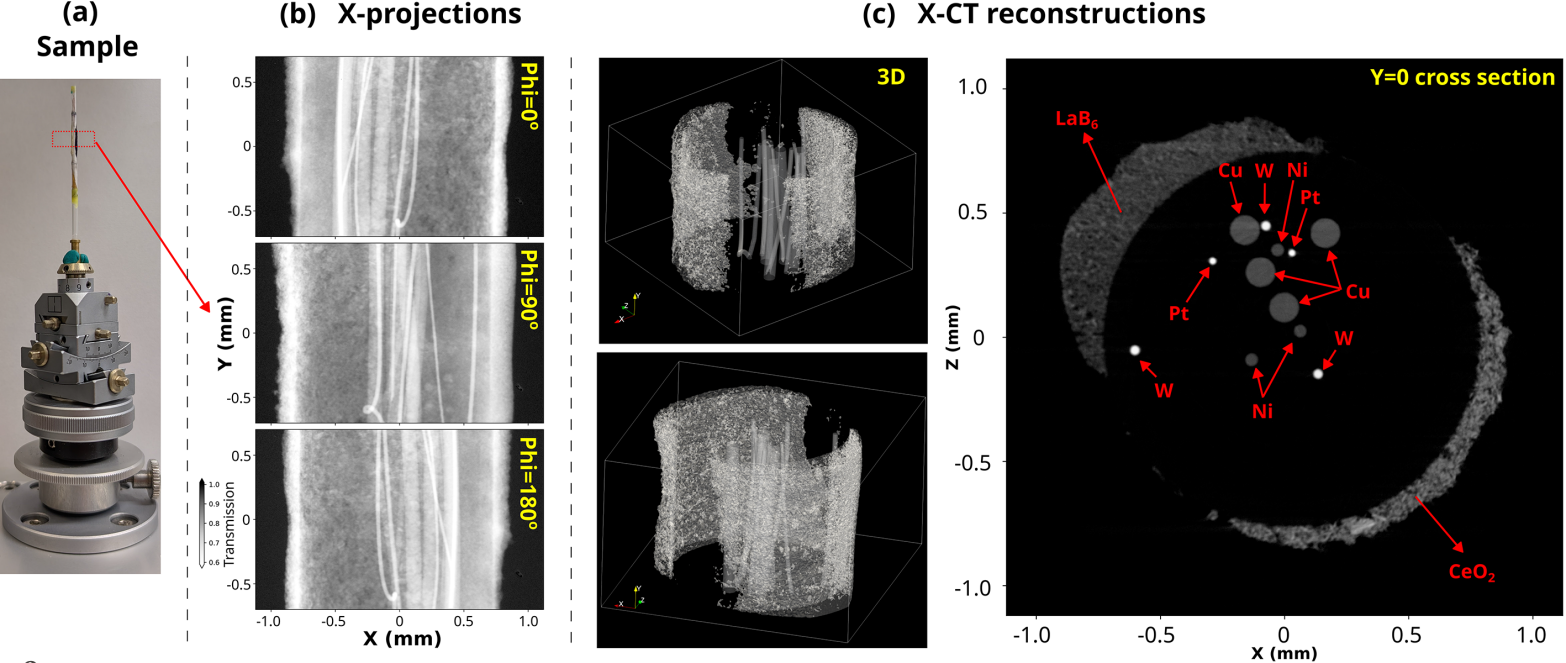
(*a*) Photograph of the demonstration sample used in this study. The dashed red rectangle shows the approximate location of the sample probed by the X-rays. (*b*) X-ray projections (radiographs) of the sample at three different Phi angles. (*c*) Reconstructed 3D X-CT volume of the sample and one *Y* = 0 cross section of the 3D reconstructed volume. The constituents of the sample are highlighted by the arrows. Quartz and Al_2_O_3_ regions cannot be identified in this 2D map.

A photograph of the demonstration sample is presented in Fig. 2 ▸(*a*). The sample was mounted on top of a Huber goniometer head to facilitate precise alignment during measurements. The sample was prepared by inserting high-purity metal wires of platinum (Pt), tungsten (W), nickel (Ni), and copper (Cu) (GoodFellow Corp., USA) into a glassy quartz (SiO_2_) capillary (Friedrich & Dimmock Inc., USA) with an outer diameter of 1.5 mm and wall thickness of 0.1 mm. The wire diameters were approximately 25 µm, 35 µm, 50 µm, and 100 µm, respectively. The remaining space inside the quartz capillary was filled with fine powder of aluminium oxide (Al_2_O_3_, NIST Standard Reference Material 676). To introduce additional structural and compositional contrast, portions of the capillary’s exterior surface were covered with standard reference powders of lanthanum hexaboride (LaB_6_, NIST Standard Reference Material 660c) and cerium dioxide (CeO_2_, NIST Standard Reference Material 674b). This heterogeneous composite was intentionally designed to contain a variety of low and high *Z* materials to test the imaging and analytical capabilities of the tomography setup, specifically to evaluate the performance and limitations of X-CT, XRF-CT, and XRD/PDF-CT modalities at the XPD beamline. The corresponding results and comparative analyses are discussed in the following sections.

### 3.2. X-ray absorption computed tomography (X-CT)

The X-ray absorption contrast of a material is governed by three principal factors: (i) the linear attenuation coefficient (μ), (ii) the material packing density, and (iii) the object thickness (*t*). The transmission of X-rays through a material follows Beer–Lambert’s law, *I* = *I*_0_exp(−μ*t*), where *I* is the transmitted intensity, *I*_0_ is the incident intensity, μ is the material specific linear attenuation coefficient, and *t* is the distance the X-rays travel through the material. A sample transmission greater than zero is necessary for the detection of transmitted or scattered X-rays in the downstream X-ray detection system.

**Figure 3:**
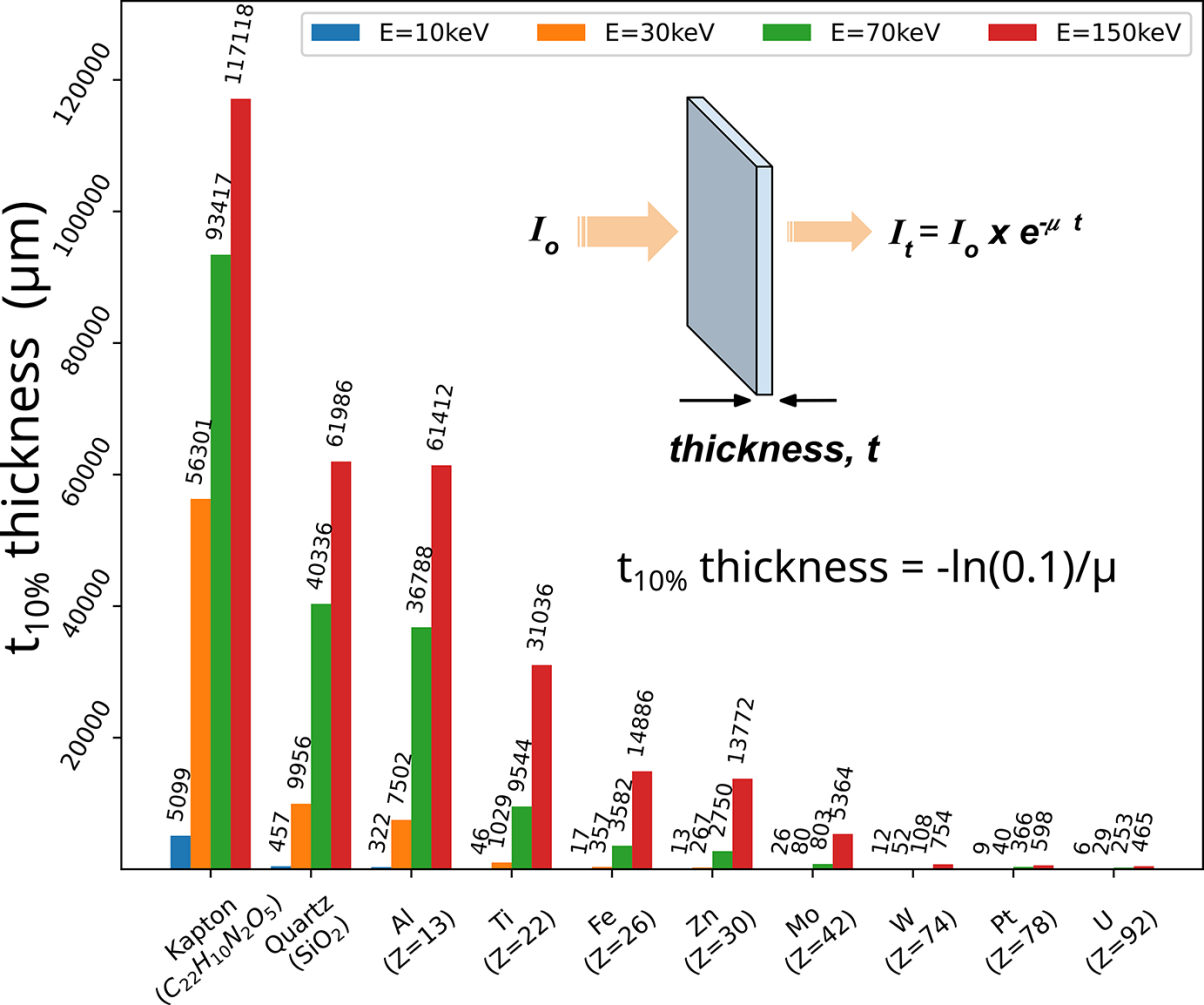
Calculated 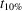 thicknesses (see text) values of several materials at 10, 30, 70, and 150 keV X-ray energies.

To provide a quantitative reference, here we define ‘10% transmission thickness’ (*t*_10%_) as the sample thickness at which transmitted intensity drops 10% of the incident beam, given by *t*_10%_ = −ln(0.1)/μ. Fig. 3 ▸ illustrates the calculated *t*_10%_ values for representative materials (Kapton, quartz, Al, Ti,…, U) over a range of X-ray energies (10, 30, 70, and 150 keV). The lower bound of 10 keV corresponds to the upper energy limit accessible at several X-ray imaging beamlines at NSLS-II, whereas 150 keV represents the highest monochromatic X-ray energy currently achievable at the HEX beamline at NSLS-II (Drakopoulos *et al.*, 2024 ▸). As shown in Fig. 3 ▸, *t*_10%_ exhibits a strong dependence on both photon energy and the atomic number (*Z*) of the material. Higher-*Z* materials, such as tungsten or uranium, demonstrate rapid attenuation at low photon energies, while higher incident energies substantially increase transmission through dense or thick samples. This analysis underscores the importance of beam energy selection in optimizing imaging contrast and penetration depth for diverse sample compositions and geometries.

At the XPD beamline, a typical experiment operates at an X-ray energy of approximately 68 keV. For comparison, a pure uranium sample exhibits a 10% X-ray transmission thickness of approximately 253 µm at 70 keV, whereas at 10 keV this limit is reduced dramatically to about 6 µm. Machining and preparing a hard dense material, like uranium, to such a small thickness presents significant challenges. These challenges are compounded for radioactive materials, which may require special precautions or where any destructive sample manipulation is prohibited. Therefore, the ability to access high photon energies is a critical advantage for synchrotron-based studies of dense and hazardous materials which are commonly encountered in nuclear materials or actinides research. At the XPD beamline, this capability enables experiments on nuclear materials to be performed safely while maintaining compliance with radiation dose constraints. Specifically, experiments can be conducted with a controlled dose rate not exceeding 100 mrem h^−1^ at a distance of 30 cm from the sample. This distinctive operational feature provides a unique opportunity to perform high-energy diffraction and tomography measurements on dense, highly radioactive, or otherwise hazardous samples that are inaccessible under conventional synchrotron facilities.

Fig. 2 ▸(*b*) presents some representative X-ray projections (radiographs) of the demonstration sample acquired at three different rotation angles (Phi). The internal metal wires enclosed within the quartz capillary, as well as the powder layers adhering to the outer surface, are clearly visible in the radiographs. In contrast, the Al_2_O_3_ powder inside the quartz capillary and the quartz wall itself exhibit minimal absorption contrast at 68 keV due to their low attenuation coefficients, rendering them nearly transparent to the incident X-rays. The reconstructed 3D X-CT volume is shown on the left side of Fig. 2 ▸(*c*), revealing the spatial distribution of the constituent materials. The loose powders of LaB_6_ and CeO_2_ are observed along the outer surface regions of the capillary. A cross-sectional slice at *Y* = 0 from the reconstructed volume [right panel of Fig. 2 ▸(*c*)] clearly displays all four embedded metal wires, which are marked by red arrows. While differentiation among the wires can be guided by their respective X-ray attenuation properties, only Pt and W are distinctly separable from Ni and Cu due to their higher absorption contrast. The limited contrast between elements of similar atomic number underscores the inherent challenge and resolution limits of the X-CT technique in discriminating compositionally similar phases.

The data presented in Figs. 2 ▸(*b*) and 2(*c*) are a result of 15 min-long X-ray tomography. The sample was continuously rotated from 0° to 180° at a rotation speed of 0.2° s^−1^, with an exposure time of 0.5 s per projection. A total of 1800 projection images were captured during fly scanning, complemented by 100 dark-field and flat-field frames. Each projection was subsequently dark-subtracted and flat-field corrected. Prior to reconstruction, artifact mitigation steps—including zinger removal and stripe removal—were performed using the *Algotom* library (Vo *et al.*, 2021 ▸). The final three-dimensional reconstructions were carried out using the filtered back projection (FBP) algorithm, yielding high-quality volumetric images suitable for quantitative analysis.

### 3.3. X-ray fluorescence computed tomography (XRF-CT)

**Figure 4:**
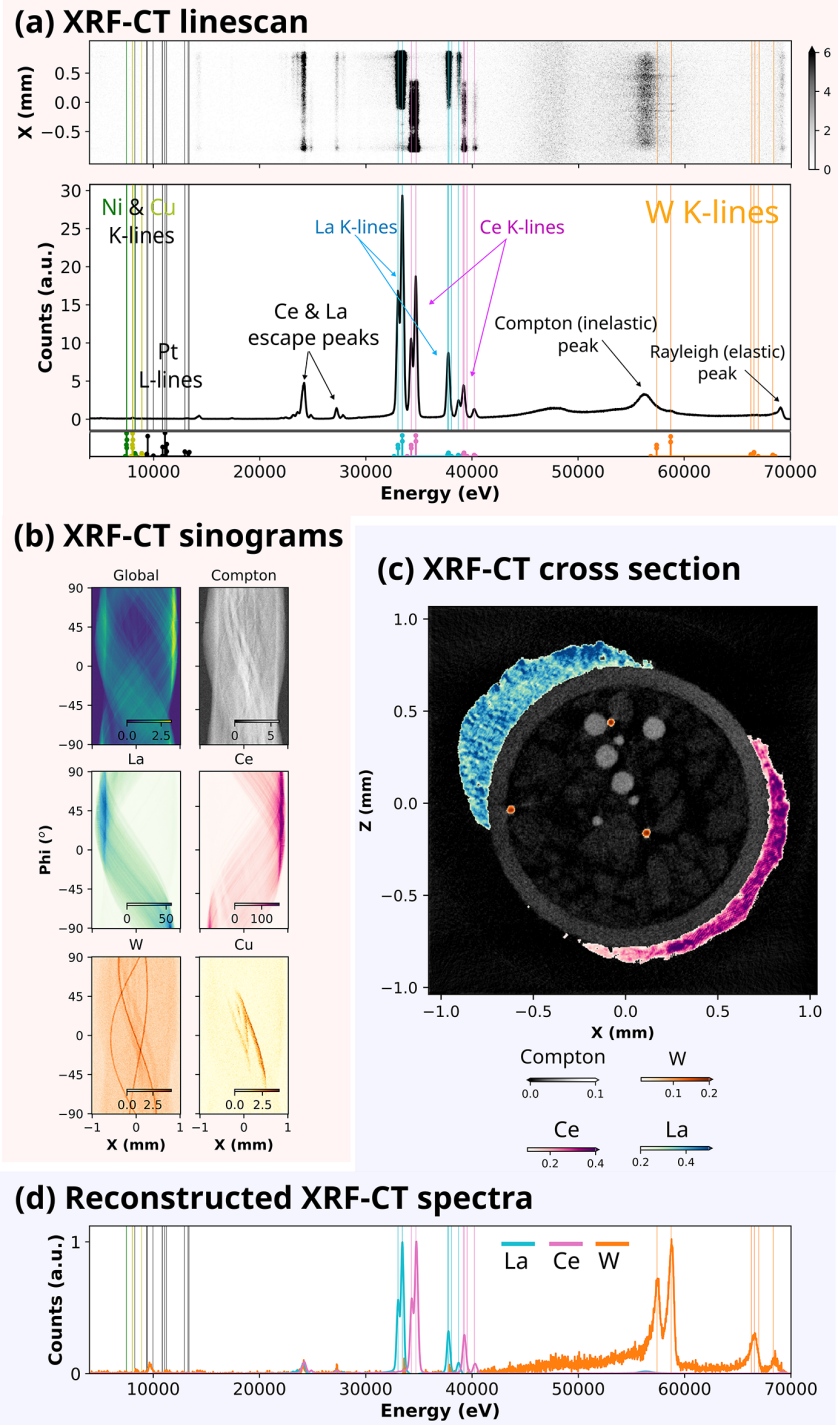
(*a*) X-ray fluorescence line-scan of the demonstration sample. Calculated XRF characteristic lines positions are indicated with the vertical lines in the bottom panel. (*b*) Total intensity (global), Compton, La, Ce, W and Cu XRF-CT sinograms. (*c*) XRF-CT *y*-cross section. La, Ce, and W reconstructions are overlayed on a Compton map. (*d*) Individual XRF spectra corresponding to La, Ce and W lines from the ROI pixels chosen from the *y*-cross section.

In contrast to X-CT, where radiographic projections generate the 2D images for tomographic reconstruction, XRF-CT relies on a point-by-point scan with a pencil beam to produce the required spatial resolution. The sample is translated through a tightly focused beam, while energy-resolved fluorescence spectra are recorded for each position, thereby constructing the projection data sequentially. The upper panel in Fig. 4 ▸(*a*) shows an X-ray fluorescence line-scan obtained along the *X*-direction for the demonstration sample shown previously in Fig. 2 ▸(*a*). Averaging this line-scan XRF signal along the *X*-dimension, one obtains an integrated XRF spectrum, as shown in the middle panel of Fig. 4 ▸(*a*). Several distinct peaks are visible in the measured spectrum. The broad feature centered at around 58 keV corresponds to the Compton scattering peak, arising from inelastic scattering processes within the sample. Adjacent to this are multiple XRF peaks associated with tungsten (W) *K*-lines. The experimentally observed peak energies show excellent agreement with theoretical transition energies, indicated by the vertical stem lines at the bottom of Fig. 4 ▸(*a*). The Rayleigh (elastic) scattering peak is observed at around 68 keV, corresponding to the energy of the incident monochromatic X-ray beam.

The most intense peaks in the averaged fluorescence spectrum in Fig. 4 ▸(*a*) correspond to lanthanum (La) and cerium (Ce) *K*-lines. Escape peaks emerging from germanium (Ge) elements of the detector are observed at ∼ 9.9 keV below the La and Ce *K*-lines. The *K*-lines of Ni and Cu elements as well as *L*-lines of Pt and W elements that are present in the sample are within the 5–10 keV energy range of the spectrum. However, the limited energy resolution of the XRF detector combined with the proximity of the Ni and Cu *K*-α and*K*-β lines complicates unambiguous elemental discrimination between these metals. Furthermore, because the incident X-ray energy (68 keV) is significantly higher than the ionization thresholds of these elements, the corresponding fluorescence cross sections are relatively low, further reducing signal intensity.

For every position along the line-scan constituting a single projection, we record a complete XRF spectrum. The measured XRF dataset has dimensions of *X*, Phi, and energy. Upon reconstruction along the energy dimension, a three dimensional data (*X*, *Z*, and energy) set is obtained. The *XZ* plane in this definition corresponds to one *Y* = 0 cross section of 3D volume shown in Fig. 2 ▸(*b*). This allows us to divide each projection into a subset of projections, one for each energy bin provided by XRF detector channels. Each sub-projection can be reconstructed individually into a tomographic slice for that energy. Thus, the tomographic slices contain local XRF spectra in each of the reconstructed pixels. Such reconstructed XRF spectra are shown in Fig. 4 ▸(*d*). On the other hand, the line integrals of the XRF intensities for each energy are not independent, due to secondary excitation, or incomplete, due to secondary absorption, and thus, the conditions for tomographic reconstruction are not perfectly satisfied. However, for non-quantitative analysis, we can neglect these effects.

Characteristic fluorescence peaks were used to construct XRF-CT sinograms, as illustrated in Fig. 4 ▸(*b*). A global sinogram was generated by averaging the entire reconstructed XRF spectrum along the energy axis, while a Compton-scattering sinogram was extracted by averaging the energy bins in the region of interest (ROI) between *E* = 55.625 keV and *E* = 57.040 keV, corresponding to a Compton peak. Similarly, La, Ce, and W sinograms were constructed based on their respective emission peak positions. Reconstruction of these sinograms yielded two-dimensional cross-sectional maps of elemental distributions within the sample [Fig. 4 ▸(*c*)]. The reconstructed Compton scattering CT image shows features consistent with those observed in the X-CT results, and effectively delineating the positions of the quartz capillary, embedded metal wires, and the outer LaB_6_/CeO_2_ domains. Reconstructions based on the W *K*-lines unambiguously determine the locations of the W wires inside the capillary. The LaB_6_ and CeO_2_ powder regions on the capillary exterior are distinctly highlighted as colored zones in Fig. 4 ▸(*c*), demonstrating the effectiveness of XRF-CT for elemental mapping of heterogeneous, multiphase materials.

The reconstructed image in Fig. 4 ▸(*c*) enables precise identification of pixel positions corresponding to individual wire components within the quartz capillary. Using this spatial information, full XRF spectra were extracted for the tungsten (W) wire and for the LaB_6_/CeO_2_ powders located along the outer surface of the capillary. The corresponding reconstructed XRF spectra are presented in Fig. 4 ▸(*d*). Notably, the reconstructed W XRF spectrum exhibits excellent agreement with the simulated *K*-edge spectrum, with Compton scattering contributions substantially reduced, thereby enhancing the relative intensity of the W *K*-line peaks. While platinum (Pt) and tungsten (W) wires, as well as LaB_6_ and CeO_2_ powders, could not be distinguished from one another in the X-CT reconstructions due to their similar absorption contrast in X-CT [as seen in Fig. 2 ▸(*c*)], XRF-CT provides a clear chemical discrimination of (i) LaB_6_ from CeO_2_, and (ii) W from other Pt/Ni/Cu wires. This demonstrates the complementary strength of fluorescence tomography in resolving compositionally similar materials that remain indistinguishable in conventional absorption-based imaging. However, XRF-CT is unable to discriminate Pt/Ni/Cu wires due to Pt *K*-lines not accessible at 68 keV X-ray energy as well as for reasons that will be discussed hereafter.

It is important to note that the XRF-CT sinograms corresponding to lower energy emission lines can be affected by secondary absorption effects, particularly in geometries with partial coverage or dense surrounding materials. Extending the tomographic rotation to full 360° could partially mitigate such artifacts. This is evident in the Cu *K*-line of the sinogram shown in Fig. 4 ▸(*b*) where signal intensity diminishes for Phi < −45° and Phi > 45°. These angular ranges correspond to orientations where the LaB_6_ or CeO_2_ domains obscure the Cu wires, therefore attenuating the Cu *K*-line XRF signal.

In the case of Al_2_O_3_, the characteristic *K*-lines of Al (1.56 keV) and oxygen (537 eV) lie at energies too low to be effectively detected due to substantial attenuation by air absorption. On the other hand, the excitation energy for the Pt *K*-line (75.73 keV) exceeds the incident energy 68 keV, preventing its excitation. Although *K*-lines fluorescence from Ni and Cu and *L*-lines of Pt are energetically permitted and weakly observed in the measured XRF spectra, the signal intensity is insufficient for constructing reliable sinogram for XRF-CT.

These results highlight some of the intrinsic limitations of XRF-CT, particularly when applied to samples containing elements with low fluorescence yields or those whose characteristic emission energies fall outside the effective excitation or detection range. The demonstration sample used here thus serves to clearly illustrate both the strengths and constraints of XRF-CT under high-energy experimental conditions.

The data presented in Fig. 4 ▸ were acquired over 160 minutes of beam time. The sample is rotated from 0 to 180° in 219 steps. At each rotation, a continuous *X*-direction line scan spanning 2.1 mm was performed in fly-scan mode. Although the sample diameter was smaller than this range, additional scanning length was included to compensate for possible off-center rotation. The *X*-translation stage operated at a linear velocity of 0.05 mm s^−1^, and the XRF detector acquisition time was set for 0.1 s per frame, corresponding to a spatial sampling interval of 5 µm per point and total 421 points along the *X*-axis. The full data set was reconstructed using the Algebraic Reconstruction Technique (ART) algorithm (Kak & Slaney, 2001 ▸) as implemented in the *tomopy* library (Gürsoy *et al.*, 2014 ▸).

### 3.4. X-ray diffraction computed tomography (XRD-CT)

Similar to XRF-CT, which relies on element-specific fluorescence signatures, XRD-CT relies on X-ray diffraction contrast to probe the crystalline structure of materials. X-ray diffraction enables the identification of crystalline phases, quantification of their relative abundances, and evaluation of structural parameters such as crystallite size and microscopic strain. At synchrotron beamlines, XRD data are typically acquired using a two-dimensional area detector, as shown in Fig. 1 ▸. Because the XRD and XRF detectors operate independently and do not interfere with each other, simultaneous collection of XRF-CT and XRD-CT data collection is possible.

**Figure 5:**
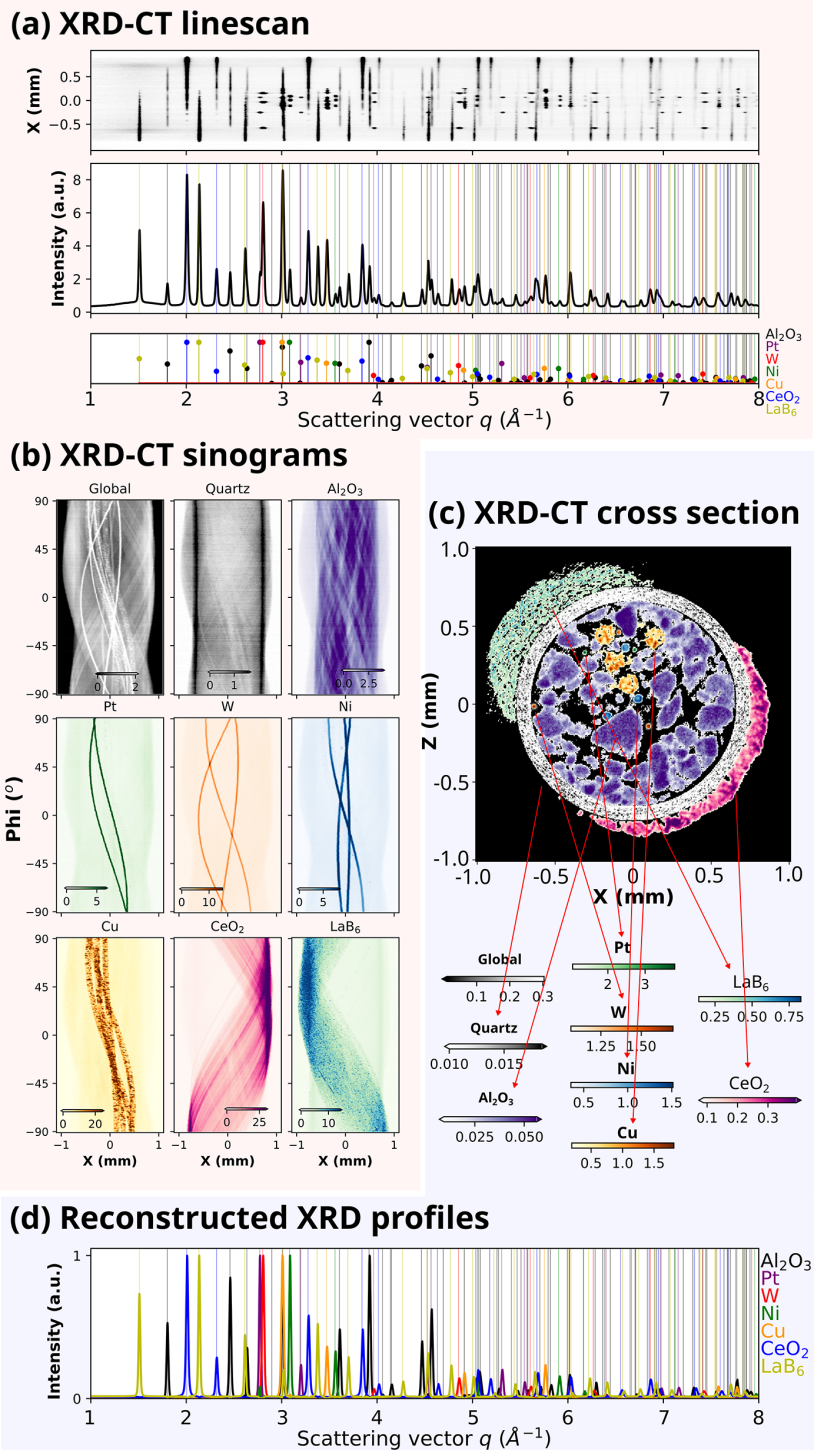
(*a*) X-ray diffraction line-scan of the demonstration sample. The top panel values are obtained by averaging along the scattering vector, *q*, axis. Simulated XRD profiles are shown by vertical lines in the bottom panel. (*b*) Global, quartz, Al_2_O_3_, W, Pt, Cu, Ni, and CeO_2_ sinograms. (*c*) Reconstructed XRD-CT *y*-cross section. Individual reconstructions are overlayed. (*d*) XRD spectra corresponding to individual phases from the pixels chosen on the *y*-cross section.

The upper panel in Fig. 5 ▸(*a*) shows an XRD line-scan of the demonstration sample, where the horizontal axis represents the scattering vector *q* and the vertical axis corresponds to the sample translation values along the *X*-direction. Averaging this dataset along the translation axis yields a one-dimensional XRD diffractogram. In contrast to the averaged XRF spectra shown in Fig. 4 ▸(*a*), the XRD pattern exhibits numerous sharp diffraction peaks, reflecting its strong sensitivity to the crystalline phases within the sample rather than merely elemental composition. Given that the constituent phases of the demonstration sample—Al_2_O_3_, Pt, W, Ni, Cu, LaB_6_, and CeO_2_—are well known, simulated diffraction profiles for these materials were generated and compared with the experimental data. The bottom panel of Fig. 5 ▸(*a*) shows the calculated diffraction patterns, which align closely with the measured XRD profile, confirming the presence of all expected crystalline phases.

The sinograms corresponding to the crystalline phases present in the demonstration sample are shown in Fig. 5 ▸(*b*). Each XRD-CT sinogram was generated using the intensity of distinct diffraction peaks representative of individual phases. For example, LaB_6_ has a distinct peak at around *q* = 1.51 Å^−1^ for the [100] reflection; therefore the LaB_6_ sinogram was constructed by averaging the *q* values in the *q* = 1.50–1.52 Å^−1^ range. In contrast to the XRF-CT sinograms in Fig. 4 ▸(*b*), for which Pt, Ni, Cu, and Al_2_O_3_ could not be effectively reconstructed, distinct XRD-CT sinograms were successfully obtained for all these phases, as shown in Fig. 5 ▸(*b*). The composite XRD-CT cross section in Fig. 5 ▸(*c*) was produced by overlaying the 2D reconstructed maps for each phase after applying a minimum threshold. Agglomerates of polycrystalline Al_2_O_3_ powder inside the quartz capillary are clearly visible. In contrast to the X-CT and XRF-CT reconstructions, the XRD-CT results reveal distinct spatial separation between Pt/W and Cu/Ni wires. Interestingly, the orange regions in Fig. 5 ▸(*c*), corresponding to Cu wires, are not cylindrical unlike X-CT or XRF-CT reconstructions, which we attribute to the presence of large grains within the Cu wire. This interpretation is supported by the non-linear pixel response observed in the corresponding Cu sinogram [Fig. 5 ▸(*b*)]. A similar observation is also present in LaB_6_ regions. It is know that such a high granularity, or single-crystal like features, give rise to line/streak artifacts in the tomograms. A filtering-based approach was developed by Vamvakeros *et al.* (2015 ▸). This observation also demonstrates that the XRD-CT can provide information about the degree of crystallinity of the materials studied. As shown in Fig. 5 ▸(*c*), LaB_6_ powders and Cu wires have larger grains compared with Al_2_O_3_ and CeO_2_ powders as present in the demonstration sample.

Similarly to XRF-CT, we can divide each recorded XRD-projection by scattering vector *q* into sub-projections, one for each *q*-bin as provided by the diffraction detector chain. Consequentially, local XRD diffractograms can be calculated for each pixel in the tomographic slice. The intensities in each *q*-bin can be regarded as being independent of each other, as required for truthful tomographic reconstruction. Multi-beam diffraction and multiple scattering are thus assumed to be negligible effects.

The generation of local diffractograms opens a powerful way to separate background and decompose individual phases present in the sample. We can reconstruct the full XRD profiles of individual wires and Al_2_O_3_, LaB_6_, CeO_2_ powders in the demonstration sample as shown in Fig. 5 ▸(*d*). Remarkably, the averaged XRD diffractogram from Fig. 5 ▸(*a*) can be decomposed into individual components [Fig. 5 ▸(*d*)] without the need for quantitative phase analysis. These 1D profiles provide a basis for further analysis, such as Rietveld refinement or peak profile fitting, to extract lattice parameters, crystallite sizes, and possible microstrain effects.

The dataset presented in Fig. 5 ▸ was collected at the same time as the XRF-CT data presented in Fig. 4 ▸, using identical translational (*X*, 421) and rotational (Phi, 219) sampling schemes. The frame acquisition time of the area detector was set as 0.1 s, as that of XRF detector. Unlike 1D XRF data that do not require computationally extensive post-processing, the raw 2D data from XRD detector requires dark-field subtraction and azimuthal integration prior to reconstructions. Azimuthal integration was performed using the *pyFAI* library (Kieffer & Wright, 2013 ▸), and the tomographic reconstructions were carried out using the ART algorithm (Kak & Slaney, 2001 ▸) as implemented in *tomopy* (Gürsoy *et al.*, 2014 ▸).

### 3.5. Pair distribution function computed tomography (PDF-CT)

**Figure 6:**
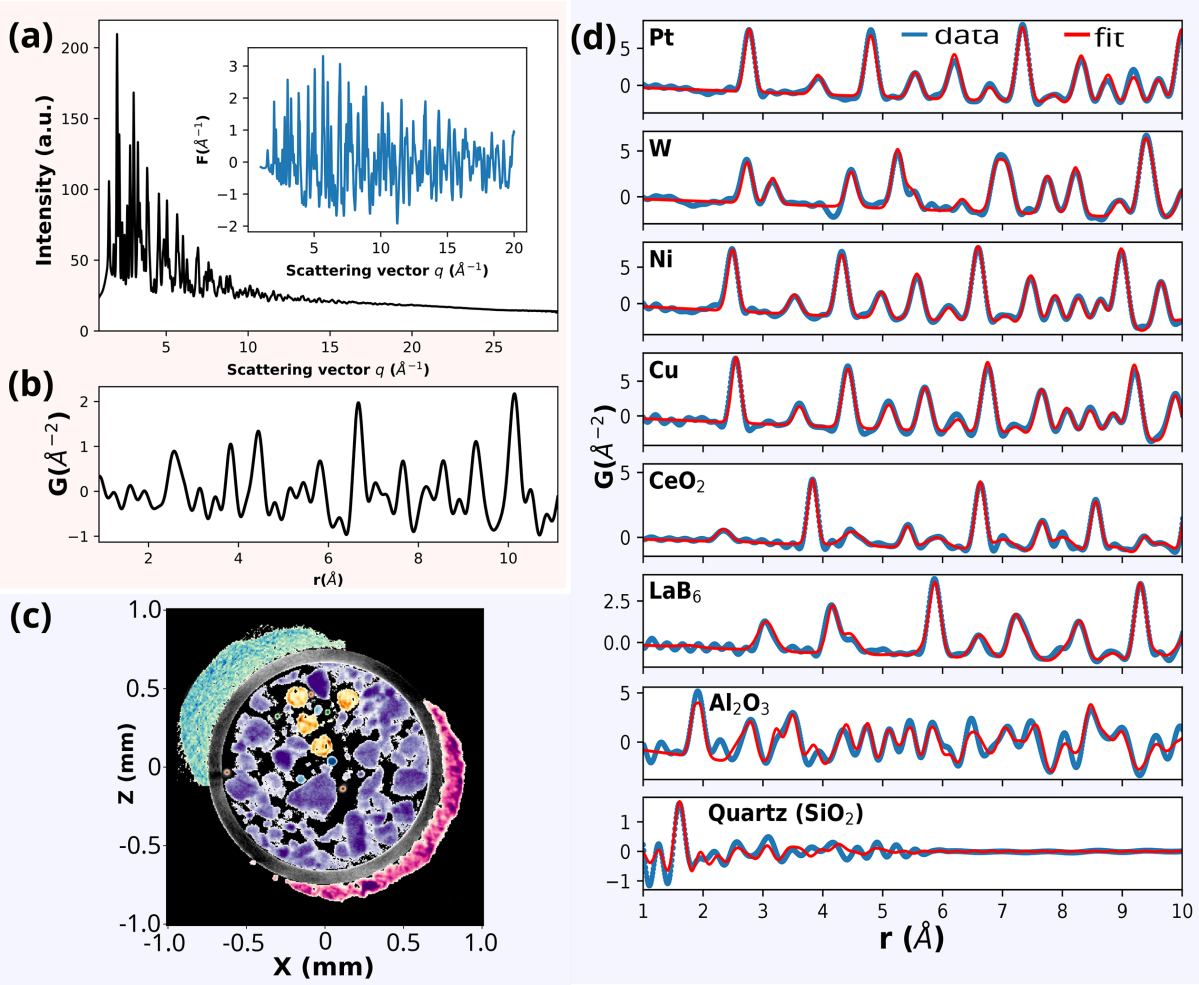
(*a*) Averaged total scattering pattern of the demonstration sample measured in PDF configuration. The inset figure shows *F*(*q*). (*b*) Pair distribution function, *G*(*r*), obtained from the sine Fourier transform of *F*(*q*). (*c*) Reconstructed PDF-CT *y*-cross section. Individual reconstructions are overlayed. (*d*) PDF-CT decomposed PDFs of the constituents of the demonstration sample with their structure model calculated *G*(*r*) fit.

The PDF-CT measurements were performed on the same demonstration sample. The total scattering data for PDF analysis were collected by placing the 2D area detector closer to the sample to obtain larger *q*-range. The sample orientation with respect to the X-ray beam in Fig. 6 ▸(*a*) is the same as Fig. 5 ▸(*a*) whereas the sample to detector distance was reduced from 650 mm to 150 mm to maximize the *q*-range (*q*_max_ = 30 Å^−1^). The detector is mounted on a motorized linear stage that has a speed of 5 mm s^−1^. Therefore, it takes approximately two minutes to switch between XRD-CT to PDF-CT modes.

In PDF analysis, judicious background subtraction and elemental normalization are applied to convert the measured X-ray scattering intensity, *I*(*q*), into the reduced structure function, *F*(*q*). This function is derived from the total scattering structure function, *S*(*q*), and is defined as *F*(*q*) =*Q*[*S*(*q*) − 1]. The real-space pair distribution function, *G*(*r*), is then obtained from *F*(*q*) through a Fourier transform (Guinier, 1994 ▸; Terban & Billinge, 2022 ▸; Egami & Billinge, 2012 ▸). The corresponding *F*(*q*) and *G*(*r*) functions are presented in the inset of Figs. 6 ▸(*a*) and 6 ▸(*b*), respectively.

The reconstructed PDF-CT cross section shown in Fig. 6 ▸(*c*) was obtained using the same procedure as for the XRD-CT reconstruction in Fig. 5 ▸(*c*), yielding similar spatial phase distributions. Fig. 6 ▸(*d*) shows the PDF-CT decomposed *G*(*r*) (blue) of each component in the demonstration sample with their calculated *G*(*r*) (red). Whereas each wire is composed of polycrystals, Al_2_O_3_ is a crystalline powder, and the quartz (SiO_2_) capillary is amorphous. A broad array of compositions is, therefore, available for study via PDF analysis, varying from sharp diffraction patterns to diffuse scattering patterns. Each material was isolated from the reconstruction and reduced to their PDF (*q*_max_ = 25, *r*-poly = 0.9) using *PDFgetX* (Juhás *et al.*, 2013 ▸) as implemented in *DiffPy* (Juhás *et al.*, 2015 ▸). It is noteworthy that each voxel within reconstruction already factorizes the data, meaning no background subtraction is required beyond judicious selection of a region of interest. In the refinement of quartz, the spherical diameter was also determined to be 5.5 Å. A Hanning window was applied between 5.5 Å and 11 Å to truncate Fourier termination ripples with a cut-off frequency proportional to *q*_min_ (0.902 Å^−1^). Each material was fitted with refinements carried out on scale, lattice parameters, isotropic thermal displacement, and correlated motion contributions (*q*_damp_ = 0.026, *q*_broad_ = 0.031). Additionally, atomic positions that did not lie on special positions were refined for Al_2_O_3_. The decomposed *G*(*r*) are in good agreement with the calculated profiles based on small weighted *R* values (*R*_w_). These results collectively demonstrate the successful implementation of PDF-CT at the beamline and highlight its capability to resolve local atomic structure in complex, highly heterogeneous systems.

### 3.6. Discussion

X-CT, XRF-CT, XRD-CT, and PDF-CT are known experimental techniques (Bleuet *et al.*, 2008 ▸; Jacques *et al.*, 2013 ▸; Vamvakeros *et al.*, 2018 ▸; Price *et al.*, 2017 ▸; Farooq *et al.*, 2024 ▸), offered at most advanced synchrotron X-ray facilities. To the best of our knowledge, this represents the first demonstration of all four different tomography techniques carried out at a synchrotron beamline on the same sample using the same setup. Remarkably, the required beam size and quality were achieved here on a wiggler-based high-energy X-ray powder diffraction beamline using an adjustable sagittally bent DLM. Precise beam tuning and flexible positioning of the beamline components enabled the combined implementation of all four techniques, some of which, including XRD-CT and PDF-CT, are not feasible in a conventional laboratory setup.

X-ray absorption contrast computed tomography (X-CT), Fig. 2 ▸, served as the primary method for the initial evaluation of the sample. Although X-CT lacks elemental and phase sensitivity, it provides superior structural information in 3D—such as sharper delineation of wire diameters and voids—due to smaller voxel size (0.69 µm). It is the fastest of the four techniques, enabling full 3D acquisition of a 2.83 mm (H) × 2.07 mm (V) volume within 15 minutes. The switch from full-field X-CT to any of other scanning-based modes is fairly quick. Therefore, we can inform subsequent XRF-CT, XRD-CT, PDF-CT studies based on X-CT results.

However, X-CT offers limited applicability for samples with unknown compositions. In such cases, XRF-CT provides complementary elemental information and can be collected concurrently with XRD-CT or PDF-CT without additional measurement time. As demonstrated in this study, XRD-CT and PDF-CT deliver essential structural and phase information. In general, XRD-CT uniquely distinguishes the crystalline phases through their distinct X-ray diffraction signatures and PDF-CT reveals the structural information from non-crystalline/amorphous phases. Another application of combined XRD-CT and X-CT is using XRD-CT to label regions in the sample with distinguishable absorption contrast, and then use X-CT to trace evolution of different components in *in situ* or *operando* measurements.

In contrast, XRF-CT, XRD-CT, and PDF-CT are significantly more time-intensive than X-CT. For instance, with the current capability of the setup, the XRD-CT dataset presented in this study required approximately 2.5 h of beam time per vertical cross section. Reconstructing a volume of 2.5 mm × 1.5 mm, as shown in Fig. 2 ▸(*c*), would require 400+ hours of data collection and 100 s of TB of storage, posing substantial challenges for data-handling capabilities. Therefore, a high-throughput and large data handling protocol is under development at XPD beamline. In this context, X-CT, XRF-CT, XRD-CT, and PDF-CT are therefore best viewed as complementary techniques. Performing all four under a single setup enables integrated acquisition of morphological, structural, elemental, and phase information. Practically, X-CT can be employed for rapid screening to identify regions of interest, which can then be targeted for XRF-CT, XRD-CT, or PDF-CT, thereby optimizing measurement time and data storage requirements.

As shown in Fig. 4 ▸, *K*-line fluorescence yields of elements such as Ce and W in the demonstration sample are significantly stronger than their *L* and *M* lines. Moreover, *L* and *M* lines of high-*Z* elements (*e.g.* Pt and W) can overlap with the *K*-lines of low-*Z* elements (*e.g.* Ni and Cu) in the low energy regions of the XRF spectrum, complicating spectral deconvolution. For rare-earth elements (La–Lu), high-energy excitation is particularly advantageous since their *K* lines (33–62 keV) are well separated from each other. Given the importance of rare-earth elements in various applications, including nuclear reactors and nuclear forensics, for example, elements such as La, Ce, Nd, and Sm are prominent fission products of U and Th—the high-energy XRF-CT becomes essential. The multimodal instrument described here, combining high-energy XRF-CT with X-CT and XRD/PDF-CT, offers powerful new capabilities for investigating complex materials in current and emerging research areas.

The PDF critically depends on the high quality of data, particularly at the high-*q* region, which is difficult to achieve in a laboratory-based instrument or low energy synchrotron setups where limited X-ray energy/flux or the detector geometry restricts *q*-range coverage. Accurate background subtraction prior to Fourier transformation is also essential for obtaining reliable *G*(*r*) profiles. As demonstrated in Fig. 6 ▸(*d*), we successfully extracted high-quality *G*(*r*) of Pt, W, Ni, and Cu wires, some of which being as small as 25 µm in diameter, from a much larger scanned cross-sectional area of approximately 2 mm in diameter. Notably, the tomographic technique employed here obviates the need for subtraction of background originating from the sample, as the spatially resolved scattering from specific phases is effectively isolated from contributions of other sample constituents.

The X-ray transmission of quartz glass (SiO_2_) with 100 µm thickness is 99.42% at 68 keV therefore making the quartz capillary used in the demonstration sample of this study almost transparent and hence not easily distinguishable by X-CT, as shown in Fig. 2 ▸. Application of XRF-CT enabled the indirect detection of quartz regions through Compton scattering as shown in Fig. 4 ▸(*c*) without elemental information. Since quartz glass is an amorphous material with no long range order, the application of XRD-CT does not provide useful information. Unlike all three techniques, PDF-CT directly reveals the presence of quartz phase in a quantitative way. As shown at the bottom of Fig. 6 ▸(*d*), we successfully extracted the unique PDF of an amorphous quartz glass phase with a major peak at *r* = 1.61 Å corresponding to the well known Si–O bond length. This is a clear demonstration of the multi-scale characterization of hierarchical structures from the atomic (Å) to the macroscopic (mm) length scales and highlights the strength of using multimodal techniques.

## 4. Conclusion

This study demonstrates the value of a multimodal computed tomography setup developed at the 28-ID-2 (XPD) beamline of the National Synchrotron Light Source II (NSLS-II). The 28-ID-2 XPD beamline is a high-energy instrument designed for *in situ* and *operando* measurements using conventional XRD and PDF techniques. The multimodal tomography capabilities not only provide complementary insights into a wide range of materials but also deliver comprehensive structural and morphological information from the same specimen. The system offers tunable beam size and high-energy operation (>60 keV), enabling detailed characterization of high-*Z* and complex materials. The instrument integrates four complementary X-ray CT modalities—absorption contrast (X-CT), fluorescence (XRF-CT), diffraction (XRD-CT), and pair distribution function (PDF-CT)—to extract morphological, structural, elemental, and phase information from the same sample. The 28-ID-2 beamline is among the few facilities worldwide capable of coupling high-energy XRF-CT with X-CT, XRD-CT, and PDF-CT, allowing detailed investigation of materials containing rare-earth and other technologically critical elements. The unique combination of imaging, structural and chemical analysis enables comprehensive assessment of heterogeneous amorphous or crystalline systems, while the flexibility of this setup makes it ideally suited for *in situ* and *operando* studies of materials synthesis, transformation, and processing.

## Figures and Tables

**Table 1:** Component specifications, providers, and positions

Component	Specs	Provider	Position in Fig. 1
X-ray imaging			
Scintillator	Gadolinium aluminium gallium garnet doped with cerium (GAGG:Ce)	Crytur, USA	H
	20 µm thickness		
Prism mirror	Al coated	Edmund Optics, USA	H
Objective lens	Infinity corrected	Edmund Optics, USA	H
	5× magnification		
	34 mm working distance		
	2 µm resolving power		
	13 µm depth of field		
Visible light sensor	4096 × 3000 pixel	Emergent Vision Technologies, USA	H
	3.45 µm pixel size		
	2.83 mm (H) × 2.07 mm (V) FOV		
	80 Hz max frame rate		
X-ray fluorescence	6 mm thick Ge	Mirion Technologies, USA	I
	10 mm active diameter		
	Cryo-cooled		
X-ray scattering	CMOS flat panel	Dexela Limited, UK	K and J
	3888 × 3072 pixel		
	29 cm × 23 cm active area		
	75 µm × 75 µm pixel size		
	26 Hz max frame rate		

## Data Availability

The data presented in this work can be provided upon reasonable request.
